# P-1565. CRISPR-Cas Systems among Clinical Klebsiella pneumoniae isolates from a Tertiary Hospital in the Philippines and its Potential Association with Antimicrobial Resistance and Hypervirulence

**DOI:** 10.1093/ofid/ofaf695.1745

**Published:** 2026-01-11

**Authors:** Christian Francisco, Stessi Marie G Geganzo, Angelo dela Tonga, Ia Marie Donna Cruz, Joana Marie Gantuangco, Jonnel B Poblete, Masahiro Suzuki, Sohei Harada, Yohei Doi

**Affiliations:** National Institutes of Health, University of the Philippines Manila, Manila, National Capital Region, Philippines; University of the Philippines Manila, Manila, National Capital Region, Philippines; National Institutes of Health, University of the Philippines Manila, Manila, National Capital Region, Philippines; Philippine General Hospital, Manila, National Capital Region, Philippines; Philippine General Hospital, Manila, National Capital Region, Philippines; Philippine General Hospital, University of the Philippines, Manila, Manila, National Capital Region, Philippines; Fujita Health University School of Medicine, Toyoake, Aichi, Japan; Toho University School of Medicine, Ota-ku, Tokyo, Japan; Fujita Health University, Aichi, Aichi, Japan

## Abstract

**Background:**

Hypervirulent and multidrug-resistant *Klebsiella pneumoniae* (KP) is a growing new public health threat. Acquisition of antimicrobial resistance (AMR) and hypervirulence (HV) genes are associated with innate microbial defense systems such as the clustered regularly interspaced short palindromic repeats and their associated proteins (CRISPR-Cas). Little is known on these associations and how it affects the spread of AMR and HV genes.

**Methods:**

Forty-nine (49) KP isolates from bloodstream infection were collected and subjected to whole genome sequencing. Corresponding draft genomes were generated using reference-based assembly. CRISPR-Cas systems were identified using CRISPRCasFinder 1.1.0. Spacers and direct repeats for isolates with CRISPR-Cas systems were characterized with CRISPRTarget and MEGA12 programs. CRISPR-Cas elements were visualized through the CRISPRStudio software. Genes related to AMR, HV, multilocus sequence typing (MLST) and capsular polysaccharide (K) typing were processed using kleborate 3.0.9.

**Results:**

Twelve isolates (24%) were found to harbor CRISPR-Cas systems, wherein 11 have complete Type I-E systems. Eight (8) of 12 isolates were from community-acquired infections (CAI). Analysis of CRISPR array elements showed that majority of the spacer sequences matched with unknown sequences and with sequences of KP phages BUCT541 and KpV2811 – both recently explored as alternative therapeutics. The consensus direct repeat sequences were observed to be partially palindromic and symmetrical. All CRISPR-Cas-positive strains contain at least one AMR gene. Four isolates were resistant to carbapenems. The presence of HV genes (*iuc*, *iro*, *rmpA*, and *rmpA2*), along with ST23 and K1, was observed to be prevalent among those with CRISPR-Cas systems.

**Conclusion:**

Isolates with Type I-E CRISPR-Cas systems are linked with HV genes, ST23, and K1 characteristics, and CAI. The prevalence of such systems in KP show the complexity of transmission of AMR and HV genes.

**Disclosures:**

Sohei Harada, MD, PhD, Denka: Honoraria|Eiken Chemical Co., Ltd.: Honoraria|MSD: Honoraria|Pfizer: Advisor/Consultant|Pfizer: Honoraria|Shionogi: Advisor/Consultant|Shionogi: Honoraria|Ushio, Inc.: Honoraria Yohei Doi, MD, PhD, GSK: Advisor/Consultant|Meiji Seika Pharma: Advisor/Consultant|Shionogi: Advisor/Consultant|Shionogi: Honoraria

